# 5,10‐methenyltetrahydrofolate synthetase deficiency: An extreme rare defect of folate metabolism in two Dutch siblings

**DOI:** 10.1002/jmd2.12409

**Published:** 2024-01-14

**Authors:** Lelde Liepina, Desiree E. C. Smith, Hidde Huidekoper, Shimriet Zeidler, Mirjam Wamelink, Marie‐Claire de Wit, Martina Wilke, George Ruijter, Jörgen Bierau, Henk J. Blom

**Affiliations:** ^1^ Department of Clinical Genetics, Center for Lysosomal and Metabolic Diseases Erasmus University Medical Center Rotterdam The Netherlands; ^2^ Department of Neurology Erasmus University Medical Center Rotterdam The Netherlands; ^3^ Metabolic Laboratory, Department of Clinical Chemistry, Amsterdam Neuroscience VU University Medical Center Amsterdam The Netherlands; ^4^ Department of Pediatrics, Center for Lysosomal and Metabolic Diseases Erasmus University Medical Center Rotterdam The Netherlands; ^5^ Department of Child Neurology Sophia Children's Hospital, Erasmus University Medical Center Rotterdam The Netherlands

**Keywords:** 5,10‐methenyltetrahydrofolate synthetase, cerebral hypomyelination, folate, MTHFS, neurometabolic

## Abstract

Two siblings, presenting with a neurometabolic phenotype, were identified with 5, 10‐methenyltetrahydrofolate synthetase (MTHFS) deficiency. Whole genome sequencing in both patients demonstrated an homozygous *MTHFS* variant NM_006441.3(*MTHFS*):c.434G > A, p.Arg145Gin, which has been described before. At baseline, both patients showed moderate hyperhomocysteinemia, decreased 5‐methyltetrahydrofolate (5MTHF), and increased 5‐formyltetrahydrofolate (5‐FTHF) in whole blood. In CSF, 5MTHF levels were in the low‐normal range and 5‐FTHF was strongly increased. In our novel enzyme assay, MTHFS activity was deficient in cultured fibroblasts in both sisters. Oral treatment was initiated with escalating dose of 5‐methyltetrahydrofolate (5MTHF) up to 12 mg and hydroxycobalamin 5 mg daily. Plasma homocysteine normalized and 5MTHF became elevated in the blood of both patients. The elevated 5FTHF levels increased further on treatment in blood and CSF. This regimen resulted in some clinical improvement of patient 1. In patient 2, the clinical benefits of 5MTHF supplementation were less obvious. It seems plausible that the alleviation of the deficient 5MTHF levels and normalization of homocysteine in blood are of some clinical benefit. On the other hand, the very high levels of 5FTHF may well be detrimental and may prompt us to decrease the dose of 5MTHF. In addition, we hypothesize that the crippled MTHFS enzyme may destabilize the purinosome, which is presumably not ameliorated by 5MTHF.

## 1 INTRODUCTION

**FIGURE 1:**
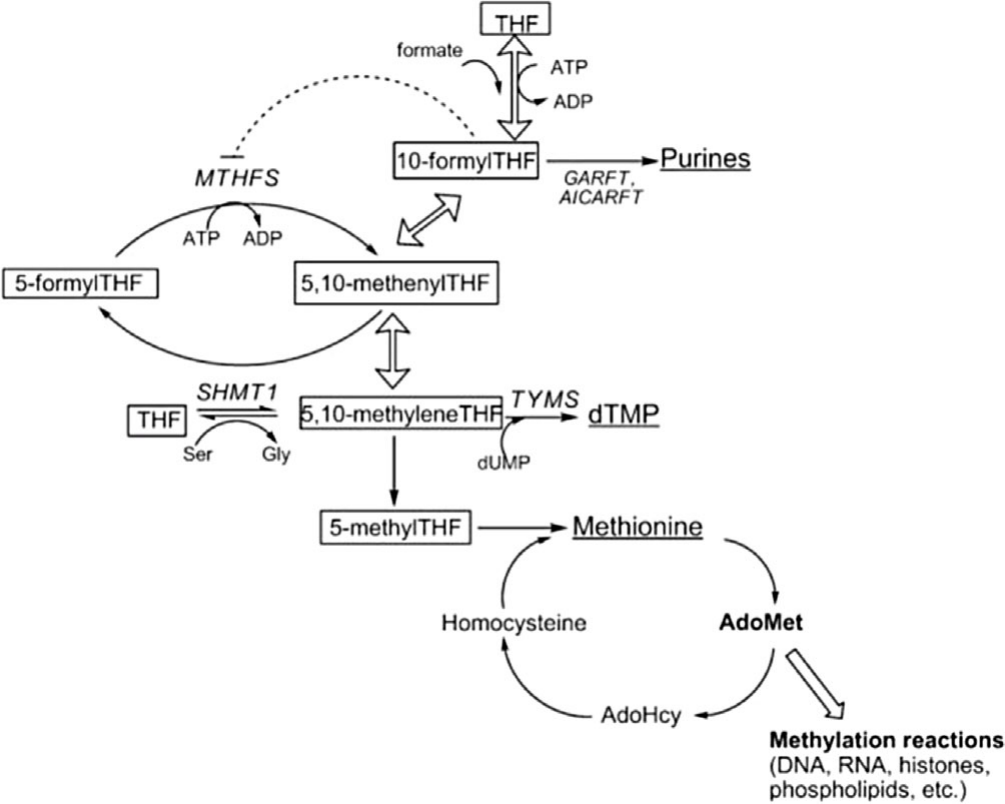
One‐carbon metabolism in the cytoplasm.^8^

5,10‐Methenyltetrahydrofolate synthetase (MTHFS) deficiency (OMIM# 618367) is an extremely rare defect of folate metabolism (Figure 1). Seven patients with MTHFS deficiency have been published.^1^, ^2^, ^3^, ^4^, ^5^ Core clinical features are microcephaly, severe global development delay, cerebral hypomyelination, epilepsy, and failure‐to‐thrive. Anemia has also been reported. In CSF, folate was low, and neurological sequela were found that in general are also observed in cerebral folate deficiency disorders. In cultured fibroblasts, accumulation of 5‐formyltetrahydrofolate (5FTHF, folinic acid), the substrate of MTHFS, was described.

We describe two cases with MTHFS deficiency, reporting on the underlying variants, and the shift in the various forms of folate in blood and CSF, and the partial clinical responsiveness to 5‐methyltetrahydrofolate (5MTHF) supplementation. We developed a novel enzyme assay of MTHFS and so we were able to demonstrate reduced MTHFS activity in cultured fibroblasts.

## 2 MATERIALS AND METHODS

### 2.1 Quantification of folate species in plasma, blood, and CSF

The various folate vitamers were determined as described before.^6^ In brief, plasma, blood, or CSF were deconjugated in ascorbic acid solutions, deproteinized, purified using folate‐binding protein affinity columns, concentrated by solid‐phase extraction (SPE), evaporated, dissolved in eluent, separated on a C18 column and determined by liquid chromatography tandem mass spectrometry (LC–MS/MS).

### 2.2 Measurement of MTHFS enzyme activity in fibroblasts

Fibroblasts were obtained by skin biopsy of the two patients and controls and were grown in DMEM medium (Invitrogen, Carlsbad, CA, USA) supplemented with 10% (v/v) heat‐inactivated fetal calf serum (Invitrogen) and 1% (v/v) penicillin–streptomycin (Invitrogen). Cultures were grown to confluency in 75 cm^2^ culture flasks (Greiner Bio One, Frickenhausen, Germany) and maintained at 37°C in an atmosphere of 5% CO_2_. Cells were harvested using trypsin (Invitrogen) and washed twice with Hank's buffered salt solution (Invitrogen). Cell pellets were stored at −80°C.

Fibroblast pellets were suspended in 200 μL 100 mmol/L TRIS/HCl buffer (pH = 7.3), and subjected to three freeze–thaw cycles. The cell suspension was subsequently centrifuged for 5 min at 10.000 g and the supernatant was used for the enzyme assay. Total protein concentration was determined using the bicinchoninic acid protein assay (Sigma, Deiselhofen, Germany).

MTHFS activity was assessed by incubating 10 μg of total protein at 37°C in 100 μL of 70 mmol/L TRIS/HCl buffer (pH = 7.3) containing 20 mmol/L MgCl_2_, 0.5 mmol/L ATP, and 5 μmol/L 5‐FTHF. After 30 min, the samples were placed directly on ice. Subsequently, 20 μL 1 μmol/L [^13^C_5_]‐5,10‐methenyltetrahydrofolate was added as internal standard, and the incubation mixture was centrifuged at 11.000 rpm at 4°C using molecular weight cutoff filters (Amicon Ultra, 10.000 MWCO). The produced 5,10‐methenyltetrahydrofolate was measured by LC–MS/MS as described before.^6^ Intra‐assay variations were 5.2%.

### 2.3 Quantification of homocysteine

Total homocysteine concentrations were measured in plasma after reduction by DTT (dithiothreitol) by LC–MS/MS (Waters Acquity UPLC coupled to Waters Xevo TQS Micro) as described in Smith D et al.^7^

## 3 RESULTS

### 3.1 Patient 1

**FIGURE 2:**
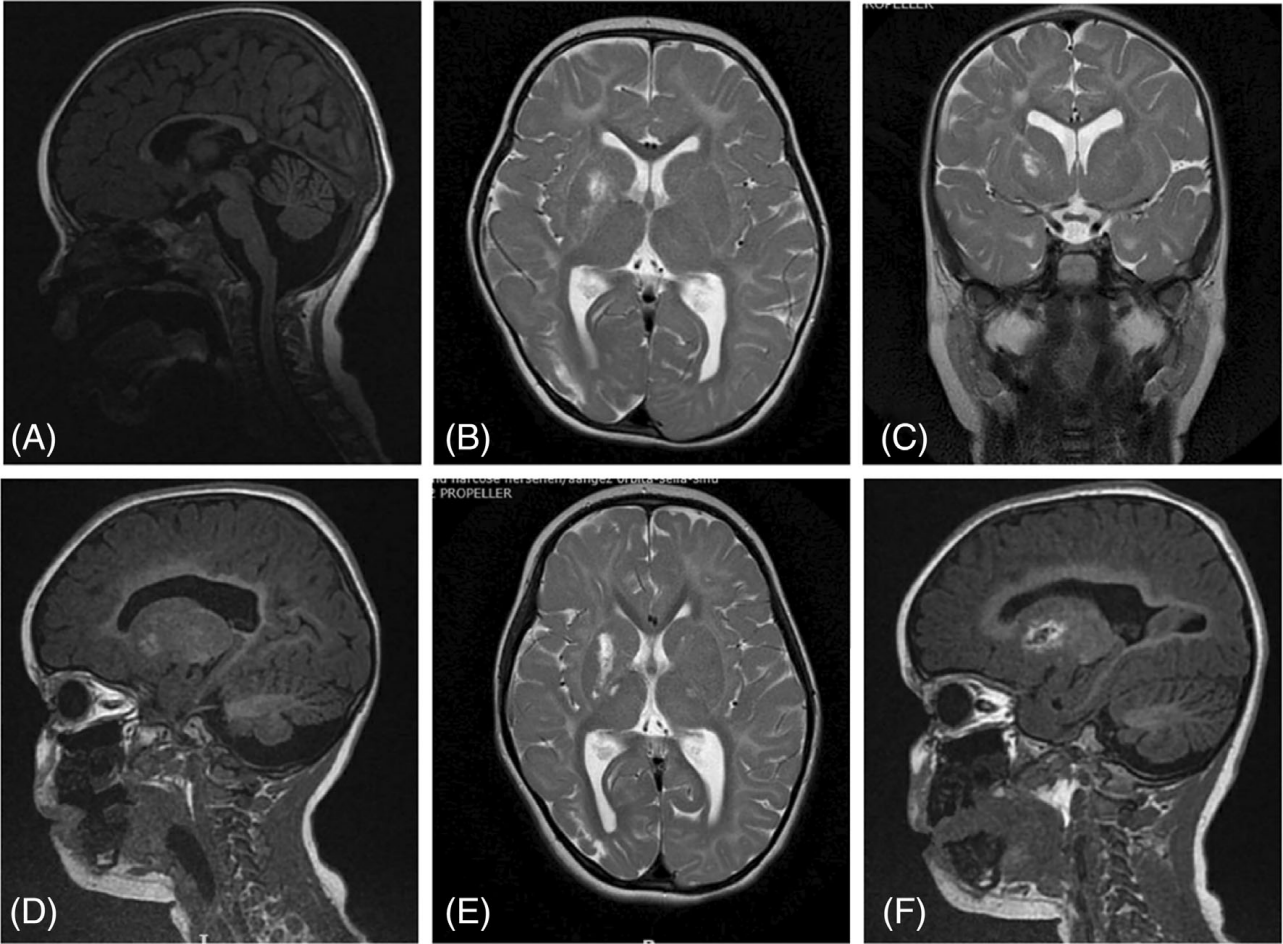
Sagittal T1 (A, D, F), axial T2 (B, E) and coronal T2 (C) images of Patient 1 at 2 years (A–C) and 4 years (D–F) of age. The initial MRI at 2 years (A–C) shows a thin corpus callosum and delayed myelination for the age – absent subcortical white matter myelination on T1‐weighted images and absent posterior limb of the internal capsule myelination on T2‐weighted images. Follow‐up MRI at the age of 4 years (D–F) demonstrates no significant increase of volume of cerebellum and vermis, no significant progress in myelination. Increased T1 weighted signal abnormalities in basal ganglia and thalamus.

Patient 1 is a 6‐year‐old female, first‐born child from non‐consanguineous parents. She was born at term with an induced labor at 42 weeks followed by an uneventful neonatal period. A smaller head circumference was seen prenatally by ultrasound, but at the birth the head circumference was within the normal range (33 cm, −1.6 SD). Head growth was slow, resulting in microcephaly at −3 SD at the age of 15 months Although her development continued to progress, moderate global developmental delay became apparent already during the first year of life prior to seizures. Feeding difficulties, constipation, and episodic vomiting led to failure to thrive in the first 2 years of life, and the start of tube feeding for which a gastrostomy was inserted at the age of 3 years. Neurological evaluation at the age of 2 years showed good social interaction, she speaks a few words. There is bulbar hypotonia with drooling, and swallowing problems, axial hypotonia with brisk tendon reflexes, and ataxia left more than right sided. She developed seizures at the age of 2.5 years. Epilepsy was initially well controlled with Levetiracetam. MRI of the brain (Figure 2), at the age of 2 years, showed cerebral hypomyelination, T1 hyperintensities in basal ganglia bilaterally, cerebellar and vermis atrophy or hypoplasia and a thin corpus callosum. MRI was repeated at the age of 4 years after 1 year of treatment with 5MTHF, and it demonstrated no significant progress in myelination as well as increased deep white matter abnormalities in basal ganglia and thalamus bilaterally (Figure 2).

SNP array analysis revealed a 19 Mb long runs of homozygosity (ROH) segment on chromosome 15. This segment includes the *MTHFS* gene. Whole exome sequencing (WES) demonstrated a homozygous *MTHFS* variant NM_006441.3(*MTHFS*):c.434G > A, p.Arg145GIn which has been previously described by Rodan et al.^1^ Both parents are heterozygous carriers of this mutation.

MTHFS deficiency was confirmed by very low MTHFS activity in cultured fibroblasts of 0.67 nmol/h/mg protein in patient 1 and 0.71 in patient 2 (reference 4.0–8.4; *n* = 6) nmol/h/mg protein. 5MTHF was reduced in whole blood, in the low‐normal range in CSF (Table 1) and high normal in plasma (51, reference 5–50 nmol/L). Concentrations of 5FTHF were markedly increased in whole blood and CSF (see also Table 1) and in plasma (data not shown). Plasma total homocysteine (tHcy) was moderately increased (17 and 31, reference 2–9 μmol/L).

**TABLE 1:** Folate concentrations in blood and CSF before and on various regimens of treatment with 5MTHF. Reference ranges 5MTHF: in blood 95–469 nmol/L and in CSF 40–140 nmol/L. 5FTHF is not detectable in blood and CSF in reference samples.

Patient 1	Treatment (5MTHF)	Blood		CSF	
Age	mg/day	5MTHF (nmol/L)	5FTHF (nmol/L)	5MTHF (nmol/L)	5FTHF (nmol/L)
2 years 9 months	No	85	51	58	15
3 years 2 months	6	1051	191	‐	‐
3 years 3 months	9	1329	213	‐	‐
3 years 4 months	12	1692	226	75	27
4 years 4 months	12	1364	173	69	30

Patient 2	Treatment (5MTHF)	Blood		CSF	
Age	mg/day	5MTHF (nmol/L)	5FTHF (nmol/L)	5MTHF (nmol/L)	5FTHF (nmol/L)
11 months	No	46	86	60	18
1 years 4 months	6	1156	205	‐	‐
1 years 5 months	9	1254	199	‐	‐
1 years 6 months	12	1747	236	85	29
2 years 7 months	12	2037	216	‐	‐

Treatment with oral 5MTHF was started using 3 g/day and increased each month via 6 and 9 up to 12 mg per day (see also Table 1). Hydroxycobalamin 5 mg daily was added to optimize the homocysteine remethylation to prevent potential accumulation of 5MTHF. The treatment resulted in normalization of plasma tHcy and blood 5MTHF. Also, in CSF 5MTHF increased, but stayed within the normal range. The elevated levels of 5FTHF before treatment increased further in blood and CSF (Table 1) and in plasma (data not shown). Within a few months on this treatment, parents subjectively reported increased alertness, more stability in a sitting position, walking with assistance, expanded vocabulary (~20 words), and she became seizure free.

Unfortunately, after a seizure‐free period of 1.5 year epileptic seizures recurred and were resistant to therapy (Levetiracetam, Vigabatrin, and Topiramate) and therefore a ketogenic diet was started. This had a clinically significant positive effect on the (secondary) generalized seizures, but she did not become seizure free.

### 3.2 Patient 2

Patient 2, the probands 2‐year‐younger sister, was born at term (42 weeks) after a normal delivery, followed by an uneventful neonatal period. Microcephaly was present at age of 4 months (−3 SD). Clinical evaluation at the age of 1 year showed mild global developmental delay, feeding difficulties and failure to thrive for which tube feeding was started just before the age of 2 years followed by the insertion of a gastrostomy at age 2.5 years. Neurologically, she had generalized hypotonia and developmental delay, although she developed milestones faster than her older sister. At the age of 10 months, she could roll over, but not sit or crawl.

**FIGURE 3:**
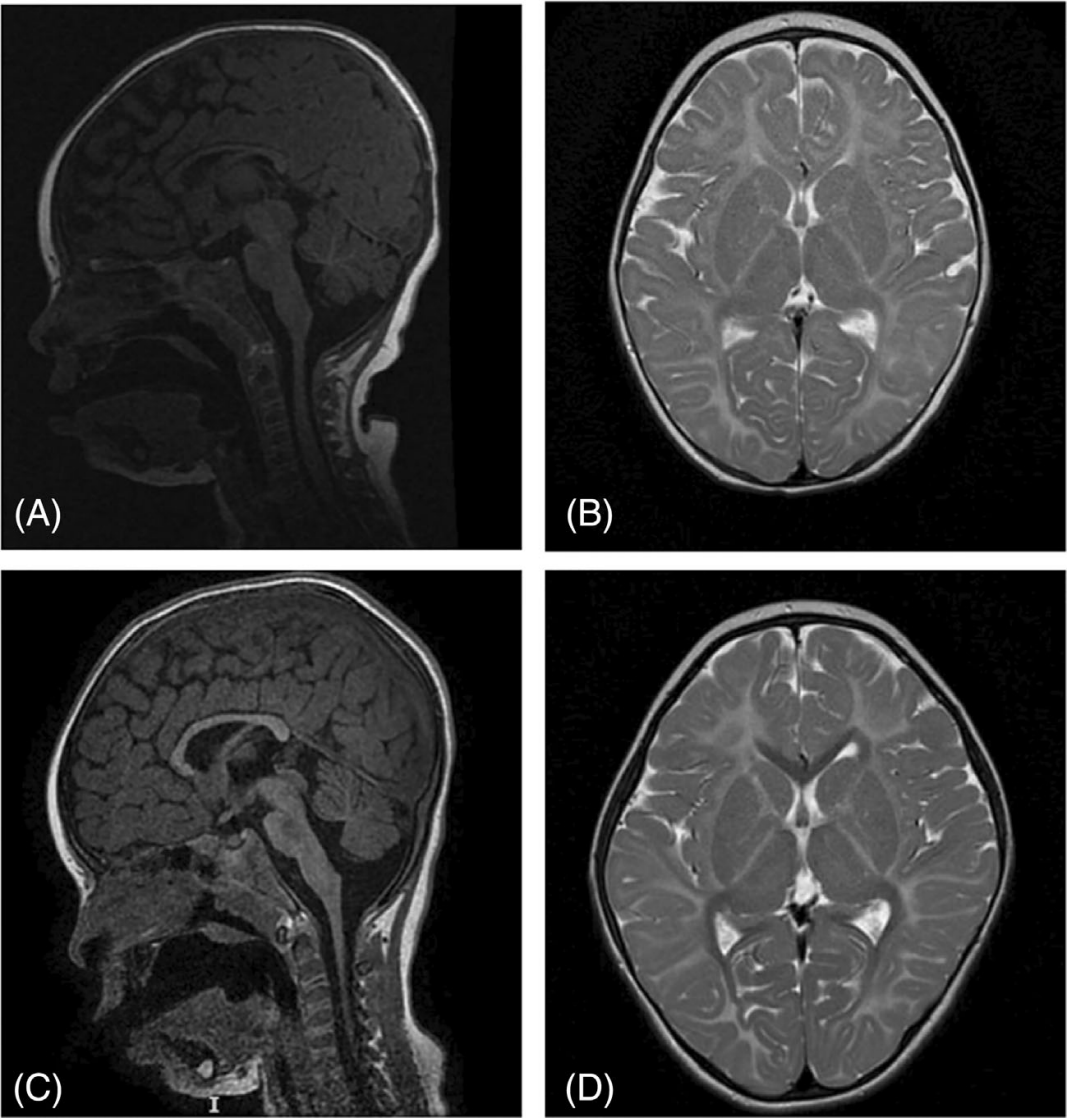
Sagittal T1 (A, C) and axial T2 (B, C) images of Patient 2 at 8 months (A, B) and 3 years (C, D) of age. Comparing the initial MRI images with the follow‐up MRI a delayed myelination for the age can be observed. By the age of 3 years white matter myelination has occurred centrally in the corpus callosum (T1‐weighted images). No significant increase of the volume of corpus callosum and cerebellum.

Brain MRI at the age of 8 months, (Figure 3) revealed cerebral hypomyelination, cerebellar and vermis atrophy or hypoplasia and a thin corpus callosum. At the age of 2.5 years, she had her first seizure and started on Levetiracetam. She was seizure‐free for a few months, but then her epilepsy became intractable as well. Considering the good experience with her sister, she commenced on ketogenic diet. Patient 2 has the same MTHFS variant as her sister. Before starting the treatment with oral 5MTHF and hydroxocobalamin, her baseline tHcy levels were increased (19 reference 2–9 μmol/L). 5MTHF was reduced in whole blood, in the low‐normal range in CSF (Table 1) and slightly increased in plasma (53, reference 5–50 nmol/L). 5FTHF was markedly increased in blood and CSF.

Treatment with oral 5MTHF and hydroxocobalamin resulted in normalization of plasma tHcy and increased 5MTHF in blood. In CSF, 5MTHF also increased to within the normal range. The previously elevated levels of 5FTHF increased further in blood and CSF (Table 1) and in plasma (data not shown).

On treatment MRI was repeated at the age of 3 years and showed no significant progress in myelination; deep white matter myelination occurred only centrally in the corpus callosum (T1 weighted images). No significant increases were observed of the volume of corpus callosum and cerebellum (Figure 3). Overall, in patient 2, the clinical benefits of 5MTHF supplementation are less obvious than in patient 1, even though she started treatment at age 1.5 years old.

## 4 DISCUSSION

Two sisters, currently 6 and 4 years of age, presented with global developmental delay, microcephaly, hypotonia, epilepsy, failure to thrive, and feeding difficulties. The diagnosis MTHFS deficiency was established in both girls by the homozygous *MTHFS* variant NM_006441.3(*MTHFS*):c.434G > A, p.Arg145GIn, markedly reduced MTHFS activity in cultured fibroblasts and major accumulations of 5FTHF in plasma, blood, and CSF. 5MTHF was deficient in whole blood and low‐normal in CSF and surprisingly slightly increased in plasma. It seems that cells are not able to retain folate in MTHFS deficiency. We have no clear explanation for this finding. One possibility maybe that 5FTHF may inhibit folylpolyglutamate synthetase. This would prevent polyglutamation of folates, which is required to retain folates inside the cells.

Seven cases with MTHFS deficiency have been described before with a comparable but not very specific neurometabolic phenotype.^1^, ^2^, ^3^, ^4^, ^5^

Folate serves as cofactor in the provision of one‐carbon units for the synthesis of purines, thymidine, and the remethylation of homocysteine to methionine (Figure 1).^8^, ^9^, ^10^, ^11^ MTHFS (5,10‐methenyltetrahydrofolate synthetase or the systematic name 5‐formyltetrahydrofolate cyclo‐ligase) catalyzes the irreversible conversion of 5FTHF to 5,10‐methenyltetrahydrofolate. The latter compound can be rapidly converted to 10‐formytetrahydrofolate (10FTHF), which donates one‐carbon units in purines synthesis (Figure 1). 10FTHF strongly binds to and inhibits MTHFS.^8^ Via this mechanism 10FTHF regulates the amount of 5FTHF converted and so balances its own intracellular levels, which is essential for adequate purine synthesis. In line, MTHFS is considered to be part of the purinosome and supports the channeling of formyl groups to purine synthesis.^8^ Mice studies have demonstrated that MTHFS is an essential gene and mice fibroblasts with heterozygous variants in MTHFS showed a reduced capacity of purine synthesis without impairment of the thymidine synthesis.^8^

In blood and CSF folate is predominantly present as 5‐methyltetrahydrofolate (5MTHF). Other folates are usually not detectable in the circulation.^6^ For the treatment of MTHFS deficiency the use of 5FTHF (folinic acid; Leucovorin) is prohibited because this metabolite is no longer metabolized in MTHFS deficiency, and so would result in even a further accumulation of 5FTHF. Treatment with folic acid also needs to be avoided because this synthetic form of folate needs to be reduced to dihydrofolate and next to tetrahydrofolate by dihydrofolate reductase before it will be biologically available. Dihydrofolate reductase has a low capacity in humans^12^ and as a consequence unmetabolized folic acid will accumulate in the body during folic acid treatment. This should be avoided because folic acid itself is considered to inhibit various folate reactions.^7^

Treatment of the two patients described in this paper with oral 5MTHF and hydroxycobalamin resulted in normalization of plasma tHcy and in blood the deficient levels of 5MTHF increased substantially to above the normal range. We assume that these are positive findings. In CSF, the 5MTHF concentration increased only marginally, like described by Rodan et al.^1^ 5FTHF increased further in blood and CSF on treatment. One may question if the accumulated 5FTHF may have clinical adverse effects, like inhibition of folate metabolism and transport. This may prompt us to decrease the dose of 5MTHF. Another potential relevant mechanism we like to discuss is that the crippled MTHFS enzyme may destabilize the purinosome. Two reactions in the purine de novo bioynthesis pathway require 10‐formylTHF, namely the ones catalyzed by glycinamide ribonucleotide formyltransferase (GARFT) and phosphoribosylaminoimidazole carboxamide formyltransferase (AICARFT) (Figure 1), which are dependent on MTHFS. There is evidence that MTHFS regulates purine de novo biosynthesis and that it co‐localizes with the purinosome.^13^ In mice, MTHFS has been shown to be a component of the purinosome in HeLa cells in a small ubiquitin‐like modifier (SUMO) protein dependent way. In mice, MTHFS appeared to be essential because homozygote knock‐out mouse embryos were not observed. Mouse embryonic fibroblasts hemizygotic for MTHFS showed decreased de novo purine biosynthesis.^8^ We are not aware of treatment strategies to stabilize the purinosome.

On treatment the parents of patient 1 subjectively reported increased alertness, more stability in a sitting position, walking with assistance, expanded vocabulary (~20 words). After a seizure‐free period of 1.5 years with monotherapy of Levetiracetam and oral 5MTHF, she unfortunately developed drug‐resistant epilepsy. Vafaee‐Shahi et al. reported a 7.5 years girl with MTHFS deficiency, who showed T1 hypeintensities in basal ganglia,^5^ comparable to our patient 1 despite the treatment with 5MTHF. Taken together treatment only marginally improved the clinical conditions of both patients. On the other hand, treatment may have prevented further clinical deterioration of both patients.

In conclusion, MTHFS deficiency is a severe neurometabolic disorder with no specific clinical symptoms. Next to MTHFS gene analysis, diagnosis relies on determination of folates in blood and MTHFS enzyme activity in cultured fibroblasts. Oral 5MTHF resulted in a clear biochemical response, but the clinical response is less obvious. The neurological damage occurring before treatment (possibly even intra uterine) may be largely irreversible. Future studies should explore additional treatment strategies in MTHFS deficiency.

## FUNDING INFORMATION

The authors received no funding.

## CONFLICT OF INTEREST STATEMENT

The authors declare no conflicts of interest.

## ETHICS STATEMENT

Written consent for publication was obtained from patients' parents.

## INFORMED CONSENT

All procedures followed were in accordance with the ethical standards of the responsible committee on human experimentation (institutional and national) and with the Helsinki Declaration of 1975, as revised in 2000. Informed consent was obtained from all patients for being included in the study; proof is available on request.

## Data Availability

Relevant data is included in the article as much as possible. Further inquiries can be directed to the corresponding author.
